# Supplementary material for: FvMYB79 Positively Regulates Strawberry Fruit Softening via Transcriptional Activation of FvPME38
Source: Int J Mol Sci. 2021 Dec 22;23(1):101. doi: 10.3390/ijms23010101 (PMC8744888; doi:10.3390/ijms23010101)
Supplement: Supplementary file 1 [file ijms-23-00101-s001.zip › supplemental figures.pdf]

Supplemental Figure S1

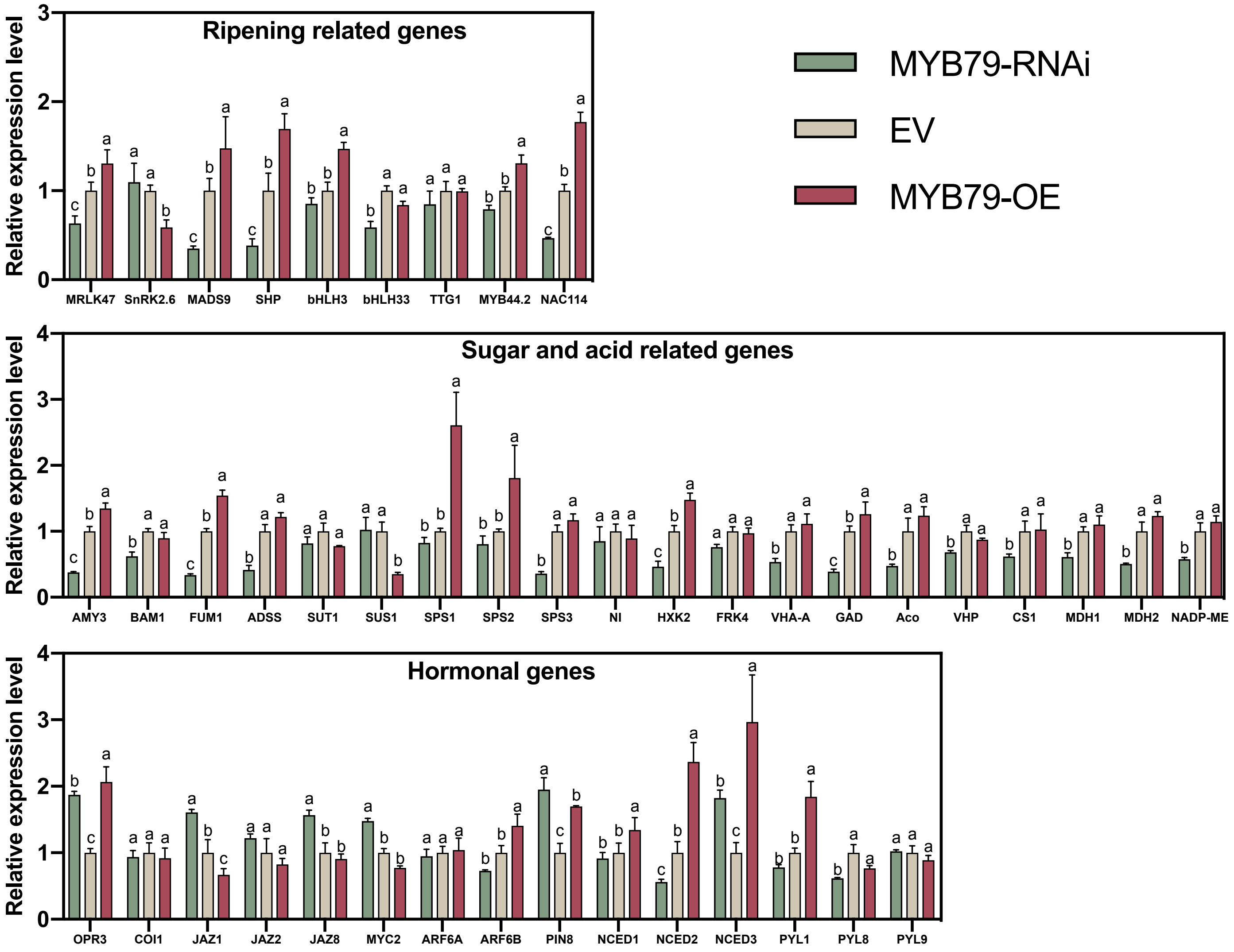

Figure S1 Expression assay in *FvMYB79* transient transgenic fruits.

Supplemental Figure S2

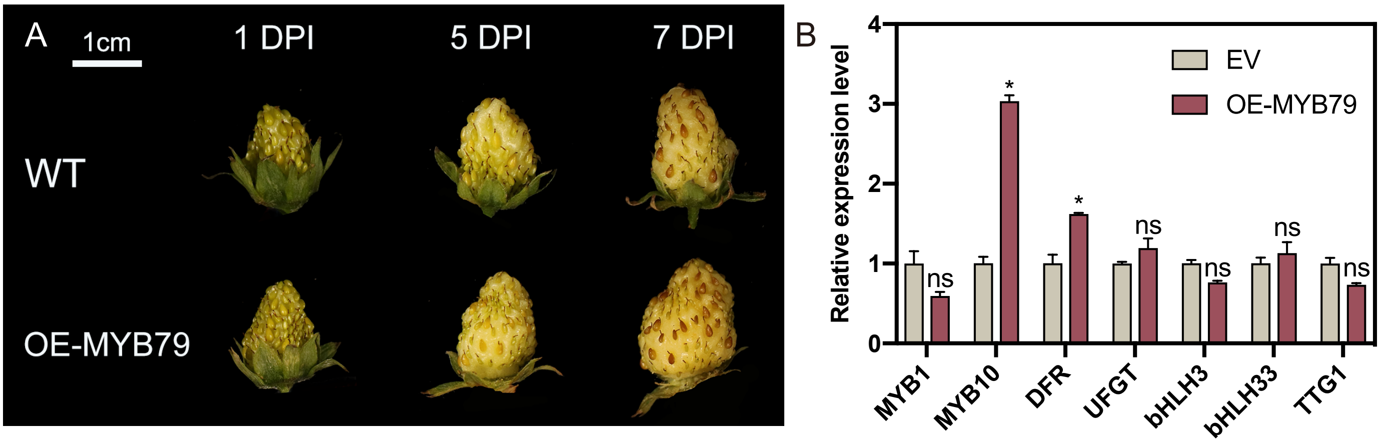

Figure S2 Overexpression of *FvMYB79* cannot promote anthocyanin biosynthesis in ‘Hawaii 4’ fruit.

(A) Phenotypes of fruits were agro-infiltrated with *FvMYB79* overexpression construct in ‘Hawaii 4’ fruit. DAI, day after infiltration; OE, overexpression.

(B) Expression assay of anthocyanin biosynthesis related genes in fruits of transient overexpression of *FvMYB79*. Relative expression levels of each gene were normalized to internal control *Fvactin*. Error bars represent SD of three independent replicates.

Supplemental Figure 3

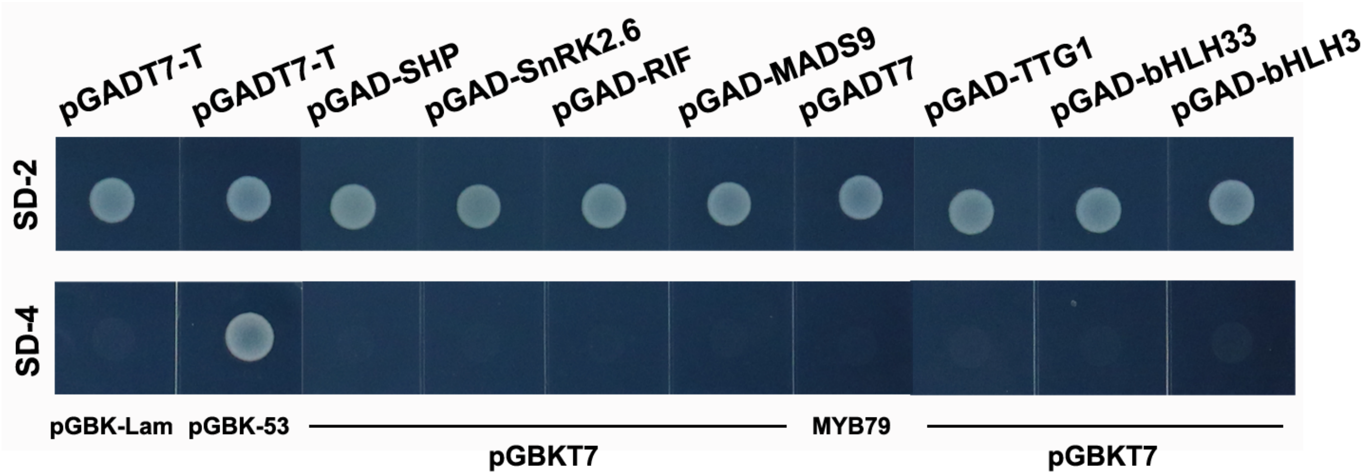

Figure S3 Yeast-two-hybrid assay

Yeast strains were dotted on SD/-Trp-Leu or SD/-Trp-Leu-His-Ade medium. BK 53-AD T interaction: positive control; BK Lam-AD T interaction: negative control.
